# Curcumin-Induced DNA Demethylation in Human Gastric Cancer Cells Is Mediated by the DNA-Damage Response Pathway

**DOI:** 10.1155/2020/2543504

**Published:** 2020-06-17

**Authors:** Ruiying Tong, Xian Wu, Ying Liu, Yang Liu, Jigang Zhou, Xinying Jiang, Li Zhang, Xiaoying He, Libing Ma

**Affiliations:** School of Life Science and Technology, Inner Mongolia University of Science and Technology, Baotou 014010, China

## Abstract

Curcumin, a natural polyphenol antioxidant extracted from the root of turmeric (*Curcuma longa*), can induce apoptosis and DNA demethylation in several types of cancer cells. However, the mechanism of its anticancer potentials and DNA demethylation effects and the potential relationships between these outcomes have not been clearly elucidated. In the present study, the effects of curcumin on the proliferation, colony formation, and migration of human gastric cancer cells (hGCCs) were explored. Reactive oxygen species (ROS) levels, mitochondrial damage, DNA damage, and apoptosis of curcumin-treated hGCCs were analyzed. Changes in the expression of several genes related to DNA damage repair, the p53 pathway, cell cycle, and DNA methylation following curcumin treatment were also evaluated. We observed that curcumin inhibited the proliferation, colony formation, and migration of hGCCs in a dose- and time-dependent fashion. A high concentration of curcumin elevated ROS levels and triggered mitochondrial damage, DNA damage, and apoptosis of hGCCs. Further, curcumin-induced DNA demethylation of hGCCs was mediated by the damaged DNA repair-p53-p21/GADD45A-cyclin/CDK-Rb/E2F-DNMT1 axis. We propose that the anticancer effect of curcumin could largely be attributed to its prooxidative effect at high concentrations and ROS elevation in cancer cells. Moreover, we present a novel mechanism by which curcumin induces DNA demethylation of hGCCs, suggesting the need to further investigate the demethylation mechanisms of other DNA hypomethylating drugs.

## 1. Introduction

Epigenetic modification can control gene expression, and malignant transformation of a cell is usually accompanied by genome-wide abnormal epigenetic modifications. DNA methylation, a stable epigenetic mark of gene repression, is known to be abnormal in all forms of cancers [1]. In cancer cell genomes, abnormal DNA methylation is usually represented by global hypomethylation along with focal hypermethylation at promoter CpG islands (CGIs) of a number of tumor suppressor genes [2]. The malignant transformation of normal cells may be partially attributed to hypermethylation at promoters and aberrant expression of some tumor suppressor genes [3, 4]. Synthetic DNA hypomethylating drugs, such as 5-azacytidine, 5-aza-2′-deoxycytidine, zebularine, and others, are used in cancer treatments. These drugs are able to decrease the levels of genomic DNA methylation and reactive tumor suppression genes and, thereby, help reverse the malignant state of cancer cells [2].

Numerous natural products extracted from plants exhibit anticancer activities [5]. Curcumin, isolated from the root of turmeric (*Curcuma longa*), can inhibit the proliferation and induce the apoptosis of several types of cancer cells [6–8]. Several underlying molecular mechanisms have been proposed for these anticancer effects, including promotion of specific microRNA expression [6, 9], inhibition/activation of specific signaling pathways [6, 7, 10, 11], reactive oxygen species (ROS)-dependent cytotoxicity [12], and induction of autophagy [13]. Further, curcumin could exert its effects on the epigenetic modification of cancer cells as summarized recently [14]. Several studies have shown that curcumin treatment could result in global genomic DNA hypomethylation, promoter demethylation, and reactivation of some tumor suppress genes (*NrF2*, *WIF-1*, *PTEN*, and *RARβ2*); these studies have also suggested that this demethylation potential of curcumin may contribute to its overall anticancer effects [15–20]. The mechanism of curcumin-induced DNA demethylation, however, has not been clearly elucidated. Moreover, the relationship between DNA demethylation and apoptosis needs to be further addressed in cancer cells.

In the present study, the effect of different concentrations of curcumin on the proliferation, clone formation, migration, and apoptosis of human gastric cancer cells (hGCCs) was determined. We explored the mechanism of curcumin-induced apoptosis and the effect of curcumin on the level of DNA methylation in hGCCs. Importantly, potential relationships between apoptosis and DNA demethylation were investigated in these cells. Our present study provides evidence that curcumin-induced high ROS levels trigger subsequent mitochondrial damage, DNA damage, and apoptosis of hGCCs and that curcumin-induced DNA demethylation of hGCCs is mediated by the DNA-damage response (DDR) pathway.

## 2. Materials and Methods

### 2.1. *In Vitro* Cell Culture

hGCC line MGC-803 was obtained from Fenghui Biotechnology Co., Ltd. (Changsha, Hunan, China) and cultured in RPMI-1640 medium (Gibco, Thermo Fisher Scientific, Waltham, MA, USA) supplemented with 10% (*v*/*v*) fetal bovine serum (FBS) (Gibco), 100 IU/mL penicillin, and 100 *μ*g/mL streptomycin at 37°C in a humidified atmosphere of 5% CO_2_. At 80–90% confluency, cells were subcultured or frozen into aliquots in 10% (*v*/*v*) DMSO, 20% (*v*/*v*) FBS, and 70% (*v*/*v*) RPMI-1640 medium and stored in nitrogen.

### 2.2. Cell Viability Assay

The effect of curcumin on the proliferation of hGCCs was evaluated using an MTT (thiazolyl blue tetrazolium bromide) assay. Briefly, cells were seeded at a density of 10^4^ cells/well in 96-well culture plates and cultured overnight to allow attachment. Then, cells were cultured in a medium supplemented with different concentrations (0, 5, 10, 15, 20, 40, and 60 *μ*M) of curcumin (Sigma-Aldrich, Merck Life Science Co., Ltd., Shanghai, China). The culture medium was replaced every 24 h. After 24, 48, and 72 h of curcumin treatment, 10 *μ*L of MTT (Sigma-Aldrich) solution, 5 mg/mL dissolved in phosphate-buffered saline (PBS), was added into each well, and cells were cultured for another 3 h. After the supernatant was removed, 150 *μ*L of DMSO was added into each well, and the plates were slightly oscillated for 10 min. Subsequently, the optical density (OD) of each well at 570 nm was recorded using a microplate reader (Synergy HT, BioTek Instruments, Inc., Winooski, VT, USA). The cell viability (%) was calculated using the following formula: (OD_570_ of the experimental samples/OD_570_ of the control samples) × 100%.

### 2.3. Colony Formation Assay

The cytotoxicity of curcumin on hGCCs was evaluated by a colony formation assay. Briefly, cells were seeded at a density of 10^3^ cells/well in 6-well culture plates and cultured overnight, then treated with different concentrations (0, 10, 15, 20, 40, and 60 *μ*M) of curcumin for 24 h. Subsequently, the cells were cultured in a medium without curcumin until clones could be clearly observed. Clones were fixed with 4% (*w*/*v*) paraformaldehyde (Sigma-Aldrich), stained with 0.1% (*w*/*v*) crystal violet solution, washed with PBS, and photographed (Ti-U, Nikon, Tokyo, Japan). The number of clones was counted, and the colony formation efficiency (%) was calculated as follows: (the number of clones/the number of seeded cells) × 100%.

### 2.4. Scratch Wound Healing Assay

The effect of curcumin on the migration of hGCCs was evaluated by a scratch wound healing assay. Briefly, 2 × 10^4^ cells were seeded in a 35 mm culture dish. When cells reached 90% confluency, a straight artificial scratch was created by a 20 *μ*L sterile tip. Then, the cells were cultured in the medium with different concentrations (0, 10, 20, 40, and 60 *μ*M) of curcumin, and the medium was refreshed every 24 h. Cells spreading into the wound area were observed and photographed every 24 h until the scratch wound completely healed.

### 2.5. Apoptosis Assay

The effect of curcumin on the apoptosis of hGCCs was evaluated by flow cytometry. Briefly, 6 × 10^4^ cells were seeded in a 100 mm culture dish and cultured overnight. Then, the cells were treated with different concentrations (0, 20, 40, and 60 *μ*M) of curcumin for 24, 48, or 72 h. Subsequently, the cells were harvested and treated with a PE Annexin V Apoptosis Detection Kit I (BD Biosciences, San Jose, CA, USA). The apoptotic cells were analyzed using a flow cytometer (FACSAria II, BD Biosciences).

### 2.6. Determination of ROS Generation

The generation of ROS in curcumin-treated hGCCs was determined using 2′,7′-dichlorofluorescein diacetate (DCFH-DA) (Sigma-Aldrich). Briefly, cells were cultured overnight at a density of 10^4^ cells/well in a 96-well culture plate and treated with different concentrations (0, 10, 20, 40, and 60 *μ*M) of curcumin for 4 or 8 h. DCFH-DA was added into the culture medium to a final concentration of 10 *μ*M, and the cells were incubated for another 1 h. The cells were then washed with PBS, and fluorescence images were captured with an inverted fluorescence microscope (Ti-U, Nikon, Tokyo, Japan). Fluorescence intensity was measured using a microplate reader (EX/EM = 485/535 nm; Synergy HT, BioTek Instruments, Inc.).

### 2.7. Mitochondrial Membrane Potential Assay

The mitochondrial membrane potential in curcumin-treated hGCCs was determined using a mitochondrial membrane potential assay kit with JC-1 (Beyotime Biotechnology, Nanjing, Jiangshu, China). Briefly, cells were cultured in 6-well culture plates until 80–90% confluency and then treated with 0 or 20 *μ*M curcumin for 12 or 24 h. The cells were dyed with JC-1 solution at 37°C for 20 min and washed twice with JC-1 dye buffer. Fluorescence images were captured with an inverted fluorescence microscope (Ti-U, Nikon, Tokyo, Japan).

### 2.8. Immunocytochemistry

hGCCs were cultured in 35 mm culture dishes. After treatment with 20 *μ*M curcumin for 24 h, the cells were washed with PBS and fixed with 4% paraformaldehyde (*w*/*v*) (Sigma-Aldrich) for 10 min. The cells were washed thrice with cold PBS and permeabilized with 0.1% (*v*/*v*) Triton X-100 (Sigma-Aldrich) (dissolved in PBS) for 10 min, followed by three washes with PBS. Subsequently, the cells were blocked with 10% (*v*/*v*) goat serum (dissolved in PBS) (ZSGB-BIO, Beijing, China) for 30 min, incubated overnight with rabbit anti-human monoclonal antibody of phosphor-*γ*H2AX (Ser139; Cell Signaling Technology, Danvers, MA, USA) at 4°C, and washed thrice with PBS. The cells were stained in the dark with Alexa Fluor 488-conjugated goat anti-rabbit IgG (H&L) (Abcam, Cambridge, MA, USA) at room temperature for 1 h, washed three times with PBS, and stained with 1 *μ*g/mL DAPI (Sigma-Aldrich) for 1 min. After washing thrice with PBS, cells were observed under a confocal laser scanning microscope (A1R, Nikon, Tokyo, Japan).

### 2.9. RT-qPCR

The effect of curcumin treatment on the expression of genes in DDR or its downstream signal pathways was determined by reverse transcription quantitative PCR (RT-qPCR). The primers were synthesized (Genewiz, Suzhou, Jiangsu, China) and are listed in Table 1. hGCCs were treated with 0 or 20 *μ*M curcumin for 24 h, and total RNA was extracted with EasyPure RNA kit (TransGen Biotech, Beijing, China). The RT reactions were performed using TransScript One-Step gDNA Removal and cDNA Synthesis SuperMix (TransGen Biotech). The reaction mixture consisted of 1 *μ*L total RNA, 2 *μ*L Anchored Oligo (dT)_18_ primer (0.5 *μ*g/*μ*L), 20 *μ*L 2 × TS Reaction Mix, 2 *μ*L TransScript RT/RI Enzyme Mix, 2 *μ*L gDNA Remover, and 13 *μ*L RNase-free H_2_O in a total volume of 40 *μ*L. Reaction mixture was incubated at 42°C for 15 min, followed by 85°C for 5 s to inactivate the reverse transcriptase. TransStart Tip Green qPCR SuperMix (TransGen Biotech) was used for qPCR. qPCR reaction mixture consisted of 0.4 *μ*L of the products of RT reaction, 0.4 *μ*L forward primer (10 *μ*M), 0.4 *μ*L reverse primer (10 *μ*M), 10 *μ*L 2 × TransStart Tip Green qPCR SuperMix, and 8.8 *μ*L nuclease-free H_2_O in a total volume of 20 *μ*L. qPCR reaction was performed by denaturation at 95°C for 30 s, followed by 45 cycles of 95°C for 5 s and 58°C for 34 s using a real-time thermal cycler (7500 Real-Time PCR System; Applied Biosystems, Thermo Fisher Scientific). The “minus RT” and “no template” reactions were used as negative controls. The specificity of real-time PCR products was confirmed by melting curves, gel electrophoresis, and sequencing. *GAPDH* was used as endogenous control gene; the level of mRNA in curcumin-treated hGCCs was normalized to that of untreated hGCCs using the 2^-*△△*CT^ method.

### 2.10. Western Blot Assay

hGCCs were treated with curcumin (0 or 20 *μ*M) or curcumin combined with a p53 inhibitor pifithrin-*α* (PFT*α*) (10 *μ*M; Sigma-Aldrich) for 24 h. Cells were then lysed by using Cell Direct Lysis Buffer (Sunshine Biotech, Beijing, China). Total proteins from cells were separated by SDS-PAGE and transferred to a polyvinylidene fluoride membrane (Millipore, Merck Life Science Co., Ltd., Shanghai). The membrane was blocked with 5% skim milk and incubated overnight with rabbit anti-human monoclonal antibodies to phosphor-*γ*H2AX (Ser139), phosphor-p53 (Ser15), phosphor-Rb (Ser780), DNMT1, or *β*-actin (Cell Signaling Technology) at 4°C, followed by incubation with horseradish peroxidase-conjugated goat anti-rabbit IgG (H&L) (ZSGB-BIO, Beijing, China) at room temperature for 1 h. Immunoreactive protein bands on the membrane were developed with BeyoECL Plus (Beyotime Biotechnology) according to the manufacturer's protocols, and the band density was analyzed using the ImageJ software.

### 2.11. Cell Cycle Assay

The effect of curcumin on the cell cycle of hGCCs was evaluated by flow cytometry. Briefly, cells were treated with curcumin (0 or 20 *μ*M) or curcumin combined with PFT*α* (10 *μ*M) for 24 h. Cells were harvested and assessed with a Cell Cycle and Apoptosis Analysis Kit (Beyotime Biotechnology). The number of cells in different phases of the cell cycle was analyzed using a flow cytometer (FACSAria II, BD Biosciences).

### 2.12. Bisulfite Sequencing

The methylation of LINE-1 transposon (L1Hs) in hGCCs was analyzed by bisulfite sequencing. Briefly, cells were treated with curcumin (0 or 20 *μ*M) or curcumin combined with PFT*α* (10 *μ*M) for 24 or 48 h. DNA extraction and bisulfite conversion were performed using TIANamp Genomic DNA Kit and DNA Bisulfite Conversion Kit (both were purchased from TIANGEN Biotech, Beijing, China). According to the sequence of L1Hs (GenBank accession number: X58075.1), a pair of primers for methylation-specific PCR (MS PCR) was designed using Methyl Primer Express Software v1.0 (Thermo Fisher Scientific) and synthesized (Genewiz). These were as follows: forward primer 5′-TAA GGG GTT AGG GAG TTT TTT T-3′ and reverse primer 5′-TTT ATT TAT CTA TAC CCT ACC CCC A-3′. MS PCR was performed using a methylation-specific PCR kit (TIANGEN Biotech); the reaction mixture consisted of 4 *μ*L bisulfite converted DNA, 1 *μ*L forward primer (10 *μ*M), 1 *μ*L reverse primer (10 *μ*M), 1.6 *μ*L dNTPs (2.5 mM), 0.4 *μ*L MSP DNA polymerase (2.5 U/*μ*L), 2 *μ*L 10 × MSP PCR buffer, and 10 *μ*L ddH_2_O in a total volume of 20 *μ*L. MS PCR reaction was performed by denaturation at 95°C for 5 min, followed by 45 cycles of 94°C for 20 s, 58°C for 30 s and 72°C for 20 s, and a final extension at 72°C for 5 min using a thermocycler (C1000 Touch Thermal Cycler; Bio-Rad, Hercules, CA, USA). The MS PCR products were cloned into a pGM-T vector using a pGM-T Cloning Kit (TIANGEN Biotech). Ligation mixture consisted of 1 *μ*L MS PCR products, 1 *μ*L pGM-T vector (50 ng/*μ*L), 1 *μ*L T4 DNA Ligase (3 U/*μ*L), 5 *μ*L 2 × T4 DNA Rapid Ligation Buffer, and 2 *μ*L ddH_2_O in a total volume of 10 *μ*L. Ligation reaction was performed at 25°C for 10 min. Recombinant pGM-T vectors were transformed into competent TOP10 *E.coli*, and LB agar plates, containing 50 *μ*g/mL ampicillin, 50 mg/mL IPTG, and 20 mg/mL X-Gal, were used to screen for positive clones. Ten clones from each sample were identified by colony PCR and sequenced. The methylation of L1Hs was analyzed by DNAMAN software v6.0 (Lynnon Biosoft, San Ramon, CA, USA).

### 2.13. Statistical Analysis

All experiments were carried out in triplicate, except bisulfite sequencing, which was repeated five times; values are shown as the mean ± standard deviation (SD). The single factor variance analysis was used to compare the significance between groups, and the significance is shown as ^∗^*p* < 0.05, ^∗∗^*p* < 0.01, and ^∗∗∗^*p* < 0.001. Difference with *p* < 0.05 was considered statistically significant.

## 3. Results

### 3.1. Curcumin Inhibits the Proliferation, Colony Formation, and Migration of hGCCs

**Figure 1:**
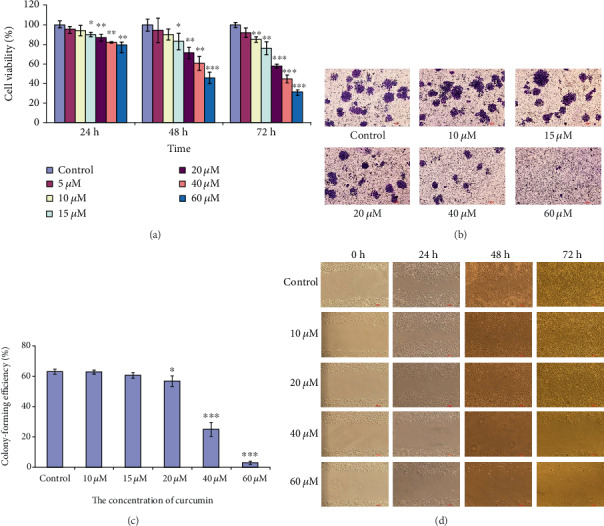
Curcumin inhibits proliferation, colony formation, and migration of hGCCs. hGCCs were treated with different concentrations of curcumin for 24, 48, and 72 h, and the proliferation, colony formation, and migration of hGCCs were assayed. (a) The cell viability was measured by MTT assay and was calculated by the following formula: cell viability (%) = (OD_570_ of the treatment samples/OD_570_ of the control samples) × 100%. (b) Cells were stained with crystal violet, the number of colonies was counted, and (c) the colony formation efficiency (%) was calculated: (the number of colonies/the number of seeded cells) × 100%. (d) Cell migration was monitored by scratch wound healing assay. All experiments were carried out in triplicates, and the data are shown as the mean ± SD. The single factor variance analysis was used to compare the significance between groups: ^∗^*p* < 0.05, ^∗∗^*p* < 0.01, and ^∗∗∗^*p* < 0.001. Difference with *p* < 0.05 was considered statistically significant.

To assess the effect of curcumin on the proliferation of hGCCs after treatment with different concentrations of curcumin, MTT assay was performed. Cell viability of hGCCs decreased with increasing concentration of curcumin after 24, 48, or 72 h incubation (Figure 1(a)). There was no significant difference in cell viability between 24 h and 48 h incubations in cells treated with 10 *μ*M curcumin; however, cell viability significantly dropped after 72 h of treatment with the same concentration of curcumin. The cell viability in the control and 10 *μ*M groups was 100 ± 2.61% and 85.16 ± 2.79%, respectively (*p* < 0.01). These results indicate that curcumin inhibited the proliferation of hGCCs in a time- and dose-dependent manner.

Given that both cell cycle arrest and cell death can decrease the cell viability, we performed a colony formation assay to determine the reproductive death of hGCCs in the presence of curcumin. Increasing the concentration of curcumin resulted in an apparent reduction in the number of hGCCs colonies (Figure 1(b)), which was confirmed by statistical analysis of colony-forming efficiency: (the number of clones/the number of seeded cells) × 100%. We observed a significant difference in colony-forming efficiency between control cells and 20 *μ*M curcumin-treated groups (*p* < 0.05) and between control cells and 40 *μ*M or 60 *μ*M curcumin-treated groups (*p* < 0.001) (Figure 1(c)). These results indicate that the decrease in the viability of curcumin-treated hGCCs may be partially attributed to curcumin-exerted cytotoxicity and subsequent cell death.

The effect of curcumin treatment on the migration of hGCCs was assessed by a scratch wound healing assay. Treatment with 20 *μ*M or higher concentrations of curcumin could apparently inhibit the migration of hGCCs (Figure 1(d)). In cells treated with more than 20 *μ*M curcumin for 48 h or 72 h, the number of the cells in an unscratched area apparently reduced and the cells showed typical apoptotic morphology, including the detachment from the bottom of culture dish and a change in cell shape (changed from an epithelial cell shape to a round-like cell shape). This result indicates that the inhibition of migration of curcumin-treated hGCCs may also be attributed to cell death.

From the results of above experiments, we concluded that 20 *μ*M or more curcumin exerted cytotoxicity on hGCCs, resulting in cell death, and this was the probable reason for the inhibitive effect of curcumin on proliferation, colony formation, and migration of hGCCs.

### 3.2. High Level of ROS Results from Curcumin Treatment, Causing Mitochondrial Damage, DNA Damage, and Apoptosis of hGCCs

**Figure 2:**
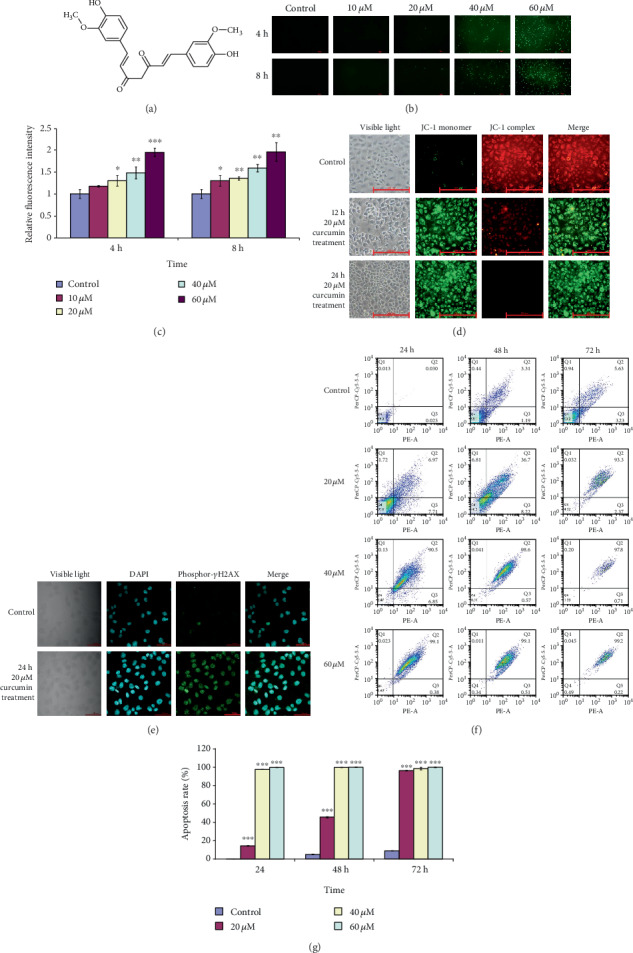
Curcumin elevates ROS levels and triggers mitochondrial damage, DNA damage, and apoptosis of hGCCs. After hGCCs were treated with different concentrations of curcumin for 24, 48, and 72 h, ROS levels, mitochondrial damage, nuclear DNA damage, and apoptosis were assayed. (a) The structure of curcumin. (b) The level of ROS was determined by a fluorescent dye of DCFH-DA, and (c) the fluorescence intensity (EX/EM = 485/535 nm) of DCFH-DA was measured. (d) The mitochondrial membrane potential (Δ*ψm*) was determined by a fluorescence probe of JC-1. (e) DNA damage was determined by immunocytochemistry of phosphor-*γ*H2AX (Ser139). (f) Apoptosis was determined by flow cytometry, and (g) the apoptosis rate (%) was calculated as follows: (the number of apoptotic cells/the number of detected cells) × 100%. All experiments were carried out in triplicate, and the data are shown as the mean ± SD. The single factor variance analysis was used to compare the significance between groups: ^∗^*p* < 0.05, ^∗∗^*p* < 0.01, and ^∗∗∗^*p* < 0.001. Difference with *p* < 0.05 was considered statistically significant.

Curcumin is a natural polyphenol antioxidant (Figure 2(a)). Several studies have shown that antioxidants (including vitamin C, vitamin E, and carotenoids) have double-edged effects on the level of intracellular ROS. For instance, low concentrations of antioxidants exert antioxidative effects, while high concentrations induce prooxidative effects [21]. Prooxidative effects of curcumin and its analogs have been reported [12, 22]. To confirm the prooxidative effects of curcumin, a fluorescent dye of DCFH-DA was used to measure the levels of ROS in curcumin-treated hGCCs. Treatment with 20 *μ*M of curcumin for 4 h significantly elevated ROS levels in hGCCs (Figure 2(b)). Curcumin increased the levels of ROS in a dose-dependent manner; however, prolonged treatment time did not cause further increase in ROS levels. The curcumin-driven elevated ROS levels were confirmed by comparing the fluorescence intensities of DCFH-DA-stained curcumin-treated and control hGCCs (Figure 2(c)). This result indicates that a high concentration of curcumin had a prooxidative effect, resulting in the elevation of ROS levels and subsequent oxidative stress in hGCCs, events that likely contributed to the cytotoxicity of curcumin and the death of hGCCs.

High levels of ROS can cause oxidative damage of the mitochondria producing pore formation in the outer mitochondrial membrane and a decrease in the mitochondrial transmembrane potential (Δ*ψm*), with sequential DNA damage and apoptotic cell death [23]. To assess the effect of curcumin treatment on mitochondrial oxidative damage of hGCCs, a fluorescent probe, JC-1, was used to detect the Δ*ψm*. JC-1 selectively enters mitochondria with high Δ*ψm*, spontaneously forming a red fluorescent complex; however, at low Δ*ψm*, JC-1 remains in the cytoplasm in its green fluorescent monomeric form. Compared with untreated control cells, treatment of hGCCs with 20 *μ*M curcumin for 12 or 24 h resulted in significantly higher green fluorescence that increased gradually with prolonged treatment time (Figure 2(d)). In contrast, the intensity of red fluorescence decreased with time. This result indicates that curcumin treatment caused oxidative damage of the mitochondria in hGCCs, exhibited by a decrease in the Δ*ψm*.

To determine whether curcumin treatment could cause DNA damage in hGCCs, immunocytochemistry experiments were performed to detect the phosphorylation of histone *γ*H2AX in curcumin-treated hGCCs. The phosphorylation of histone *γ*H2AX at serine 139 is a sensitive marker of double-strand breaks in DNA [24, 25]. There was a lack of fluorescence of phosphor-*γ*H2AX (Ser139) in hGCCs in control groups; however, fluorescence was detected in the nuclei of curcumin-treated hGCCs (Figure 2(e)). Moreover, as shown in the results of the following experiments, curcumin-induced DNA damage in hGCCs was further confirmed by upregulation in the expression of some DNA damage-related genes, including DNA-activated, catalytic subunit (*DNA-PK*), ATM serine/threonine kinase (*ATM*), ATR serine/threonine kinase (*ATR*), *HDAC1*, *p21*, and DNA damage inducible alpha (*GADD45A*), as well as the increase in the amount of phosphor-*γ*H2AX (Ser139) histone. These results indicate that the high level of ROS resulting from curcumin treatment caused DNA damage in hGCCs.

The damage in mitochondria and nuclear DNA can trigger programmed cell death (apoptosis) [23, 26]. After treatment with 20, 40, or 60 *μ*M curcumin for 24, 48, or 72 h, flow cytometry was used to determine the apoptosis rate of hGCCs. The apoptosis rates of hGCCs in curcumin-treated groups were significantly higher than those in control groups and increased in a dose- and time-dependent manner (Figures 2(f) and 2(g)). This result indicates that the high level of ROS resulted from curcumin treatment ultimately caused the apoptosis of hGCCs.

Therefore, high concentrations of curcumin could elevate the level of ROS, cause damage to the mitochondria and genomic DNA, and ultimately lead to the apoptosis of hGCCs.

### 3.3. Curcumin-Driven DNA Damage Triggers DDR

**Figure 3:**
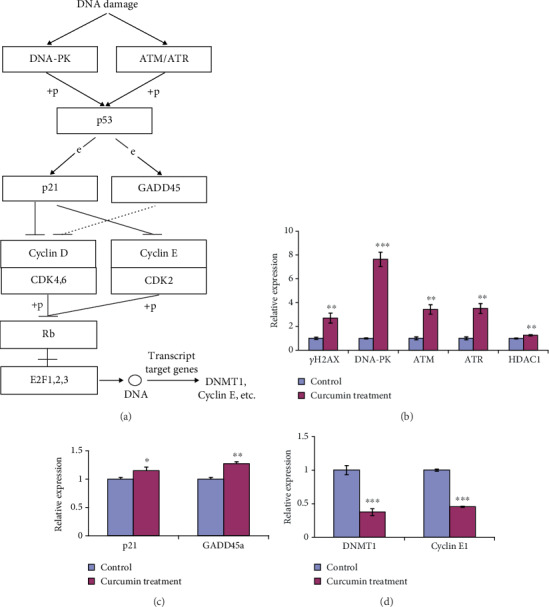
DNA damage triggers DDR and downstream signaling pathways. hGCCs were treated with 20 *μ*M curcumin for 24 h, and the expression of several genes in DDR or downstream signaling pathways was determined by RT-qPCR. (a) DNA damage-triggered DDR and downstream signaling pathways. (b) DDR-related gene expression. (c) Expression of p53 target genes. (d) Expression of E2F target genes. All experiments were carried out in triplicate, and the data are shown as the mean ± SD. The single factor variance analysis was used to compare the significance between groups as follows: ^∗^*p* < 0.05, ^∗∗^*p* < 0.01, and ^∗∗∗^*p* < 0.001. Difference with *p* < 0.05 was considered statistically significant.

The accumulation of DNA damage can trigger a DDR mechanism to repair damaged DNA or induce cell cycle arrest and/or apoptosis if damage is irreparable [27, 28] (Figure 3(a)).

We asked whether the activation of the DDR could be triggered by curcumin-induced DNA damage. Thus, the expression of several genes related to DNA repair was assessed using RT-qPCR. ATM/ATR/DNA-PK-mediated phosphorylation of serine-139 of the histone *γ*H2AX on chromatin flanking double strain break sites is characteristic of DDR [27]. Moreover, HDAC1 and HDAC2 also function in DDR to promote DNA nonhomologous end joining [29]. Treatment of hGCCs with 20 *μ*M curcumin for 24 h led to a significant increase in the expression of *ATM*, *ATR*, *DNA-PK*, *HDAC1*, and *γH2AX* (Figure 3(b)). This result confirmed the accumulation of phosphor-*γ*H2AX on the DNA in curcumin-treated hGCCs (Figure 2(e)) and further confirmed that high levels of curcumin caused DNA damage in hGCCs.

p53 is a key player in the intrinsic cellular responses to DNA damage, and the activation of p53 by phosphorylation of serine-15 can upregulate the expression of GADD45A and p21 [27, 30] (Figure 3(a)). The expression of *p21* and *GADD45A*, the target genes of p53, significantly increased after hGCCs were treated with 20 *μ*M curcumin for 24 h (Figure 3(c)). The relative expression of *GADD45A* and *p21* in treated cells was 1.27 ± 0.04 (*p* < 0.01) and 1.15 ± 0.06 (*p* < 0.05), respectively. This result indicates that curcumin-caused DNA damage could activate the p53 pathway, resulting in the upregulation of p53 target genes.

It is well known that p21 can bind to CDKs, inhibit their kinase activity, prevent the phosphorylation of Rb by cyclin E-CDK2 or cyclin D-CDK4 complexes, and arrest the cell cycle in G0/G1 phase [31, 32]. Moreover, nonphosphorylated Rb can associate with E2F factors and inhibit the expression of E2F-regulated genes, including *Cyclin E* and *DNMT1* [32, 33] (Figure 3(a)). Curcumin treatment significantly downregulated the expression of *DNMT1* and *Cyclin E1* in hGCCs (Figure 3(d)). The relative expression of *DNMT1* and *Cyclin E1* in treated cells was 0.38 ± 0.05 (*p* < 0.001) and 0.45 ± 0.01 (*p* < 0.001), respectively. These results indicate that the upregulated expression of p21 and GADD45A resulted in the abrogation of E2F target genes.

Taken together, curcumin-induced DNA damage triggered DDR and ultimately repressed the expression of *DNMT1* and *Cyclin E*, likely via the damaged DNA repair-p53-p21/GADD45A-cyclin/CDK-Rb/E2F-DNMT1/Cyclin E axis.

### 3.4. Curcumin-Induced Genomic DNA Demethylation of hGCCs Is Mediated by DDR

**Figure 4:**
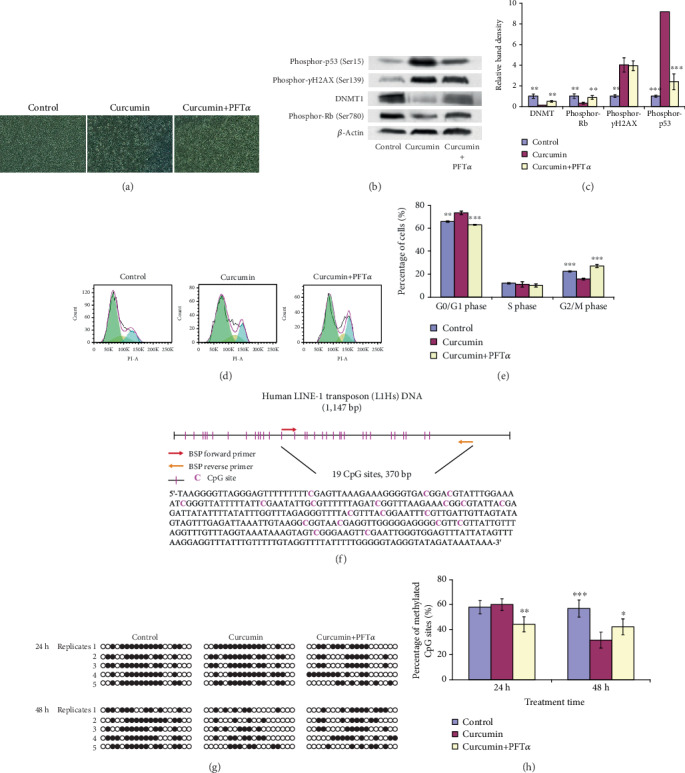
Curcumin-induced cell cycle arrest and DNA demethylation are mediated by DDR. hGCCs were treated with 20 *μ*M curcumin (or combined with 10 *μ*M PFT*α*, a p53 specific inhibitor) for 24 or 48 h, and the amount of proteins related to DDR or its downstream signaling pathways, the cell cycle, and DNA methylation was determined. (a) PFT*α* treatment alleviated apoptosis of hGCCs caused by curcumin treatment (exhibited by the decrease in the number of round-shape, apoptosis cells). (b and c) The amount of phosphorylated *γ*H2AX, phosphorylated p53, phosphorylated Rb, DNMT1, and *β*-actin (endogenous control protein) was determined by western blot, and the band density was analyzed by the ImageJ software. (d and e) The number of cells in different phases of the cell cycle was determined by flow cytometry. (f) One of the CGIs in L1Hs, which contained 370 bp and 19 of CpG sites, was amplified in methylation-specific PCR. (g) The methylation of this CGI was determined by bisulfite sequencing. Methylated and nonmethylated CpG sites are shown as black dots and cycle dots, respectively. (h) The percentage of methylated CpG sites was calculated as follows: (the number of methylated CpG sites/the number of detected CpG sites) × 100%. All experiments were carried out in triplicates; bisulfite sequencing was repeated five times. The data are shown as the mean ± SD. The single factor variance analysis was used to compare the significance between groups as: ^∗^*p* < 0.05, ^∗∗^*p* < 0.01, and ^∗∗∗^*p* < 0.001. Difference with *p* < 0.05 was considered statistically significant.

To determine whether the downregulation of *DNMT1* in curcumin-treated hGCCs was mediated by the damaged DNA repair-p53-p21/GADD45A-cyclin/CDK-Rb/E2F-DNMT1/Cyclin E axis, a p53-specific inhibitor, PFT*α*, was used in this study. PFT*α* treatment alleviated the apoptosis of hGCCs caused by curcumin treatment, as exhibited by the decrease in the number of round-shape, apoptotic cells (Figure 4(a)). As illustrated by western blotting analysis in Figures 4(b) and 4(c), treatment of hGCCs with 20 *μ*M curcumin for 24 h resulted in a significant increase in the amount of phosphorylated *γ*H2AX (*p* < 0.01) and phosphorylated p53 (*p* < 0.001). There was also a significant decrease in phosphorylated Rb (*p* < 0.01) and DNMT1 (*p* < 0.01). However, treatment with 20 *μ*M curcumin combined with 10 *μ*M PFT*α* for 24 h partially restored the changes in the amount of phosphorylated p53 (*p* < 0.001), phosphorylated Rb (*p* < 0.01), and DNMT1 (*p* < 0.01). This result confirms that curcumin treatment activated the DDR and p53 pathway, consequently repressing the phosphorylation of Rb and decreasing the amount of DNMT1. Moreover, inhibiting p53, a link in the damaged DNA repair-p53-p21/GADD45A-cyclin/CDK-Rb/E2F-DNMT1/Cyclin E axis, partially restored the abundance of DNMT1, which further confirmed the contribution of this axis to curcumin-induced decrease of *DNMT1* in hGCCs.

As discussed above, DDR can lead to cell cycle arrest, which is thought to increase the time available for DNA repair before replication or mitosis ensues [27]. Using flow cytometry, compared to control cells, curcumin treatment led to more cells staying in the G0/G1 phase (*p* < 0.01), characteristic of cell cycle arrest. PFT*α* treatment relieved the arrest exerted by curcumin treatment (*p* < 0.001) (Figures 4(d) and 4(e)), confirming that that cell cycle arrest induced by curcumin treatment was also mediated by the damaged DNA repair-p53-p21/GADD45A-cyclin/CDK-Rb/E2F axis.

To explore whether curcumin-induced downregulation in the expression of *DNMT1* leads to the demethylation of genomic DNA in hGCCs, cells were treated with 20 *μ*M curcumin for 24 or 48 h and evaluated for the methylation of L1Hs, a surrogate marker of global DNA methylation [34], using bisulfite sequencing. One of the CGIs in L1Hs contained 370 bp and 19 CpG sites (Figure 4(f)). After treatment for 24 h, the percentage of methylated CpG sites was comparable with that of control groups (*p* > 0.05). However, prolonging treatment time to 48 h resulted in a significant decrease in the percentage of methylated CpG sites (*p* < 0.001, Figures 4(g) and 4(h)). After 24 h curcumin treatment, the lack of apparent change in the level of DNA methylation was likely attributed to cell cycle arrest, with few cells undergoing DNA replication and subsequent mitosis; however, prolonging treatment time to 48 h allowed more cells to complete a cell cycle; therefore, newly synthesized DNA experienced demethylation due to the decrease in the expression of *DNMT1*. Moreover, treatment with 20 *μ*M curcumin combined with 10 *μ*M PFT*α* for 24 h resulted in a significant decrease in DNA methylation in hGCCs (*p* < 0.01, Figures 4(g) and 4(h)), corresponding to a relief of cell cycle arrest. Importantly, prolonging the combined treatment (curcumin + PFT*α*) to 48 h resulted in levels intermediate of control and curcumin-treated responses, although significant (*p* < 0.05). The observation suggests that curcumin-induced DNA demethylation in hGCCs was also mediated by the damaged DNA repair-p53-p21/GADD45A-cyclin/CDK-Rb/E2F-DNMT1/Cyclin E axis, and blocking a link in this axis relieved curcumin-induced DNA demethylation.

Together, curcumin-induced cell cycle arrest and genomic DNA demethylation were mediated by the DNA damage repair-p53-p21/GADD45A-cyclin/CDK-Rb/E2F-DNMT1/Cyclin E axis; blocking of this axis relieved cell cycle arrest and genomic DNA demethylation exerted by curcumin treatment.

## 4. Discussion

Cells can generate ROS, intermediate oxygen radicals carrying metabolites with or without an unpaired electron, during normal cellular metabolism. ROS can be overproduced when cells experience various environmental stresses, such as UV radiation or ozone exposure [21]. It has been proposed that low levels of ROS may be beneficial for normal physiological actions; however, high levels of ROS become deleterious, exhibiting pathophysiological actions [35]. The endogenous antioxidative defense system in a cell, including superoxide dismutase, catalase, glutathione peroxidase, and glutathione, can eliminate excess ROS with the assistance of exogenous antioxidants, such as vitamin C, vitamin E, carotenoids, and polyphenols, to maintain or reestablish redox homeostasis in a cell. Just as the “two-faced” physiological effects of ROS on a cell, antioxidants also exhibit double-edged effects on intracellular ROS, which are antioxidative effects in low concentration but prooxidative at high concentrations [21]. In the present study, treatment with 20 *μ*M curcumin for 4 h resulted in a significant elevation in ROS in hGCCs, with a positive correlation between increases in concentration of curcumin and ROS production, confirming the prooxidative effect of curcumin at high concentrations. The decrease in the proliferation, colony formation, and migration of curcumin-treated hGCCs, together with an increase in apoptosis, was likely the physiological consequence of curcumin-induced ROS elevation. As described earlier, several mechanisms are proposed for the anticancer effect of curcumin; however, we propose that all of these mechanisms are the subsequent events triggered by curcumin-induced ROS elevation, similar to plasma-activated medium induction of ROS-dependent autophagy of pancreatic cancer cells [36] or the curcumin-induced DNA demethylation observed in the present study. Several studies indicate that cancer cells are more sensitive to curcumin treatment than normal cells [37]. Although cancer cells have a higher level of ROS than normal cells due to their accelerated metabolism, excessive ROS accumulation may trigger oxidative stress-induced cancer cell death [38]. Therefore, the level of ROS in cancer cells may be more sensitive to curcumin treatment than that in normal cells, as higher oxidative stress in cancer cells leads to cell death. This assumption needs to be further confirmed.

In fact, curcumin's application in cancer treatment is limited due to its low water solubility, rapid metabolism, rapid systemic elimination, and low cellular uptake [39, 40]. In the present study, curcumin was dissolved into DMSO to overcome its low water solubility; then, the stock solution (30 mM) was diluted by cell culture medium. However, high concentration of curcumin used in the present study, such as 40 and 60 *μ*M, may be unattainable in the plasma of hGCCs. This needs to be further detected.

**Figure 5:**
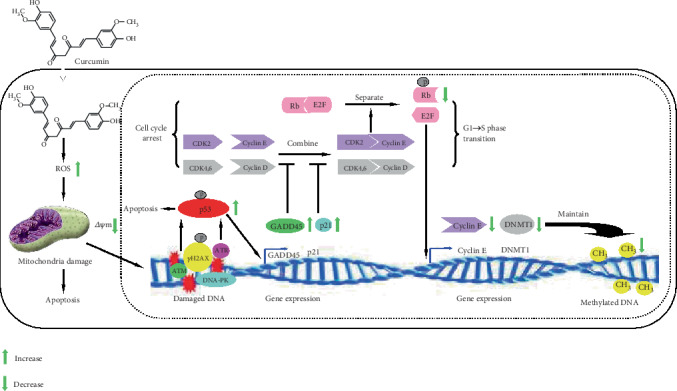
Proposed model for curcumin-induced DNA demethylation of hGCCs. Curcumin at high concentration elevates the level of intracellular ROS in hGCCs, causing mitochondrial damage (exhibited by the decrease in Δ*ψm*), DNA damage, and apoptosis. DNA damage then triggers DDR and recruits ATM/ATR/DNA-PK to strand break sites to repair damaged DNA. ATM/ATR/DNA-PK can phosphorylate both histone *γ*H2AX on chromatin flanking double strain break sites and p53, resulting in the activation of the p53 pathway. This leads to apoptosis, with upregulation of the target genes, *p21* and *GADD45A*. Expressed p21 and GADD45A may then inhibit the combination of cyclin D with CDK4,6 and cyclin E with CDK2 and prevent the phosphorylation of Rb by these cyclin/CDK complexes and, thereby, induce cell cycle arrest. Nonphosphorylated Rb may associate with E2F factors and inhibit the expression of E2F-regulated genes, including *Cyclin E* and *DNMT1*. The downregulation of DNMT1 may then lead to DNA demethylation after DNA is replicated in the S phase of the cell cycle.

High levels of ROS trigger a downstream series of cellular events, including oxidative damage of the mitochondria and nuclear DNA, as shown in the present study. These ROS-triggered cellular events may then further activate/inhibit a series of signaling pathways, such as damaged DNA repair, p53, cell cycle, and apoptosis, and upregulate/downregulate certain genes, such as *ATM*, *ATR*, *DNA-PK*, *p21*, *GADD45A*, c*yclin E1*, and *DNMT1*, ultimately leading to cell cycle arrest, apoptosis, and DNA demethylation (Figure 5). In the present study, only the expression of *DNMT1* was detected. In fact, both DNMT1 and DNMT3a are maintenance DNA methyltransferases that maintain DNA methylation and regulate synaptic function in adult forebrain neurons [41]. It needs to be further explored whether the expression of *DNMT3a* is also affected by curcumin treatment.

As we have indicated, several studies attributed the anticancer effect of curcumin to its demethylation potential and the reactivation of tumor suppression genes [15–20]. The demethylation potential of curcumin was confirmed in the present study; however, we propose that the anticancer effect of curcumin was mainly attributed to its potential for elevating the ROS level in cancer cells, as well as subsequent ROS-triggered damage of the mitochondria and nuclear DNA. The DNA damage-triggered DNA damage repair-p53-p21/GADD45A-cyclin/CDK-Rb/E2F-DNMT1/Cyclin E axis ultimately resulted in the downregulation of *DNMT1* and the demethylation of nuclear DNA (Figure 5). Interfering with the phosphorylation of p53, a link in this DNA damage repair axis, could partially restore both levels of DNMT1 and DNA methylation, further confirming the function of the axis in the modulation of *DNMT1* expression and DNA methylation. Therefore, we propose that curcumin-induced DNA demethylation was the consequence, rather than the cause, of cancer cell apoptosis. In fact, some conventional DNA hypomethylating drugs, including 5-azacytidine and decitabine, can upregulate the expression of *γH2AX* and *p21* and arrest the cell cycle of colon cancer cells in a p53-dependent fashion. The upregulation of *p21* reportedly occurs via the DNA damage/ATM/p53 axis [25]. Therefore, as shown in Figure 5, we propose a novel mechanism, by which curcumin induces DNA demethylation of hGCCs. Further studies are needed to confirm whether DNA demethylation induced by other DNA hypomethylating drugs also conforms to this mechanism.

## 5. Conclusions

Our results demonstrate that the anticancer effect of curcumin is mainly due to its prooxidative effects at high concentrations and that the oxidative stress triggers DNA damage that mediates DNA demethylation via the damage DNA repair-p53-p21/GADD45A-cyclin/CDK-Rb/E2F-DNMT1 axis.

## Figures and Tables

**Table 1:** Primers for RT-qPCR.

Primers	Sequence	Tm (°C)	Product length (bp)
ATM forward primer	5′- GAGGAGCTTGGGCCTTTGTT -3′	60.54	151
ATM reverse primer	5′- TAAGTCACGGCTGTCTGGC -3′	59.71	
ATR forward primer	5′- GGCAGTAGCTTCCTTTCGCT -3′	60.39	165
ATR reverse primer	5′- CTAATTGCACTGACTCCGGC -3′	58.99	
DNA-PK forward primer	5′- GTGGCATCCAGAGTAGCGAA -3′	59.82	165
DNA-PK reverse primer	5′- TCGCAACCAGTTCACACAGA -3′	59.82	
*γ*H2AX forward primer	5′- CGACAACAAGAAGACGCGAAT -3′	59.54	220
*γ*H2AX reverse primer	5′- CTCTTAGTACTCCTGGGAGGC -3′	58.69	
p21 forward primer	5′- GACCATGTGGACCTGTCACT -3′	59.31	176
p21 reverse primer	5′- GCGGATTAGGGCTTCCTCTT -3′	59.53	
GADD45A forward primer	5′- CCAAGGGGCTGAGTGAGTTC -3′	60.32	153
GADD45A reverse primer	5′- TCCTTCCTGCATGGTTCTTTGT -3′	60.16	
Cyclin E1 forward primer	5′- GGGTATCAGTGGTGCGACAT -3′	59.82	186
Cyclin E1 reverse primer	5′- CATGGCTTTCTTTGCTCGGG -3′	59.83	
DNMT1 forward primer	5′- GGCGGCTCAAAGATTTGGAA -3′	59.11	161
DNMT1 reverse primer	5′- CAGGTAGCCCTCCTCGGATA -3′	59.89	
HDAC1 forward primer	5′- TGCTGTTAACTACCCGCTCC -3′	59.75	187
HDAC1 reverse primer	5′- ACACTTGGCGTGTCCTTTGA -3′	60.11	
GAPDH forward primer	5′- CCATGGGGAAGGTGAAGGTC -3′	60.03	172
GAPDH reverse primer	5′- TGGAATTTGCCATGGGTGGA -3′	59.88	

## Data Availability

The raw data used to support the findings of this study are available from the corresponding author upon request.
